# A metalens-based analog computing system for ultrasonic Fourier transform calculations

**DOI:** 10.1038/s41598-022-21753-9

**Published:** 2022-10-12

**Authors:** Robert Frederik Uy, Viet Phuong Bui

**Affiliations:** 1 Hwa Chong Institution, Singapore, 269734 Singapore; 2 Electronics and Photonics Department, A*STAR Institute of High Performance Computing, Singapore, 138632 Singapore

**Keywords:** Engineering, Physics

## Abstract

Wave-based analog computing is a new computing paradigm heralded as a potentially superior alternative to existing digital computers. Currently, there are optical and low-frequency acoustic analog Fourier transformers. However, the former suffers from phase retrieval issues, and the latter is too physically bulky for integration into CMOS-compatible chips. This paper presents a solution to these problems: the Ultrasonic Fourier Transform Analog Computing System (UFT-ACS), a metalens-based analog computer that utilizes ultrasonic waves to perform Fourier transform calculations. Through wave propagation simulations on MATLAB, the UFT-ACS has been shown to calculate the Fourier transform of various input functions with a high degree of accuracy. Moreover, the optimal selection of parameters through sufficient zero padding and appropriate truncation and bandlimiting to minimize errors is also discussed.

## Introduction

The first known analog computer is the Antikythera mechanism, invented by the ancient Greeks^1^. Since then, many other mechanical and electronic analog computers have been devised to perform mathematical operations more efficiently^1–3^. Subsequently, with the development of semiconductor technology and integrated circuits, the sheer speed and reliability of digital computers eventually led to a tectonic shift in the twentieth century^2,3^. When performing complex computational tasks, however, digital computers are computationally inefficient and consume a lot of energy^2^. Unfortunately, there is little opportunity for further improvements as Moore’s law approaches its physical limits^2,4,5^. With the rising demand for ever-increasing computational capacity and efficiency^6,7^ and the recent breakthroughs in the field of metamaterials^3,8^, a new computing paradigm with very promising prospects has emerged: wave-based analog computing.

Wave-based analog computing leverages waves to perform analog computing. It has been heralded as a potential future of computing because of its high computational efficiency, low crosstalk, and powerful parallel processing^1,8,9^. Silva et al.’s seminal paper^10^ on computational metamaterials laid the foundation for other researchers to conduct studies into both optical and acoustic analog computing systems performing mathematical operations^1–4,6–35^, with some making use of the Fourier transform to do so^3,6,9,10,26–28^.

The Fourier transform (FT) is a mathematical operation that maps a function in one variable to the spectral space of its conjugate variable^14,36–38^. It is a powerful tool with wide-ranging applications in myriad disciplines^4,22–24,36–43^. Currently, the two-dimensional Fast Fourier Transform (FFT) algorithm has a computational complexity of $O({N}^{2}\mathrm{log}N)$, which is not efficient enough for certain applications, such as real-time image processing in autonomous systems^4,22–24^.

Capitalizing on the Fourier transforming property of thin lenses^36^, researchers have developed a new, analog method of performing FT calculations: the optical Fourier transform (OFT)^35,36^. The OFT, which has an apparent computational complexity of only $O(N)$, is significantly faster than the electronic FFT algorithm^22,35^. However, due to the limitations of phase modulation and phase retrieval methods^22^, it would be impractical to capture phase data when performing the OFT. Researchers thus turned to acoustic waves. Although this method is comparatively slower than the OFT, it is nevertheless faster than the FFT, and it allows for the retrieval of phase information, unlike the OFT.

Aiming to replicate the success of optical analog computing systems in acoustics, Zuo et al. developed an acoustic analog computing (AAC) system that performs FT-based spatial differentiation, integration, and convolution^26^. Several other studies on AAC systems have been conducted, but they all have an operating frequency in the kilohertz range^3,9,26–28^.

Unfortunately, performing acoustic FT at such low frequencies requires a physically bulky computing system even with the use of thin, planar metasurfaces. Therefore, researchers have sought to use ultrasonic waves instead to perform ultrasonic Fourier transform (UFT). The shorter wavelength of ultrasonic waves allows for a more compact analog computing system that is easily integrable into CMOS-compatible chips. Liu et al. developed an ultrasonic FT system without any lens^22^. This, however, would require a relatively large system as, in the absence of a lens, the UFT will only be achieved in the far field. Subsequently, Hwang, Kuo, and Lal worked on realizing the UFT with an acoustic Fresnel lens^23^, after which they used a metalens to allow for a more compact UFT computing system^24^. Besides compactness, other reasons for using a metalens include the CMOS-compatibility of materials used and ease of fabrication.

Despite the considerable work that has been done on the UFT^22–24^, there has yet to be a comprehensive study on the accuracy of the Ultrasonic Fourier Transform Analog Computing System (UFT-ACS). Thus, this study aims to fill this gap in the existing literature. Firstly, this study aims to determine how accurate the UFT’s magnitude and phase are compared with those of the analytical FT for all three types of functions, specified in Sec. 4. Unlike previous studies^22–24^, this study also takes into account the UFT’s phase, not just its magnitude. Secondly, this study also seeks to determine how to optimize the UFT calculation for space-limited functions by examining how the accuracy is affected by the level of zero padding. Thirdly, another objective of this study is to investigate the effects of truncation and bandlimiting of functions that are not space-limited and/or bandlimited on the UFT-ACS’ accuracy to allow for the optimal selection of parameters.

## Results

### The Ultrasonic Fourier Transform Analog Computing System (UFT-ACS)

**Figure 1:**
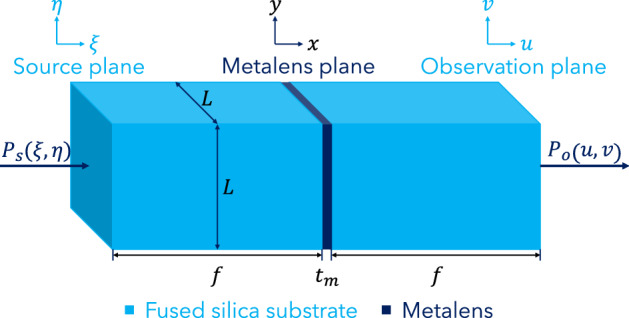
Schematic of the UFT-ACS. The figure shows a schematic of the UFT-ACS, which consists of five main parts: the input pressure field ${P}_{s}(\xi ,\eta )$ at the source plane, a substrate layer (light blue), the ultrasonic metalens (dark blue), another substrate layer (light blue), and the output pressure field ${P}_{o}(u,v)$ at the observation plane.

Referring to Fig. 1, the Ultrasonic Fourier Transform Analog Computing System (UFT-ACS) consists of five main parts: the source plane, a substrate layer, the ultrasonic metalens, another substrate layer, and the observation plane. The entire UFT-ACS has a square cross-section with side length $L$. The focal length of the metalens is $f$, which is also the thickness of both substrate layers—this is a key condition for obtaining the UFT expression. The thickness of the metalens is ${t}_{m}$.

Using concepts in acoustic wave propagation—in particular, Fresnel diffraction and lenses’ Fourier transforming property—and some approximations (see Table 1), it can be shown that the output ${P}_{o}\left(u,v\right)$ is proportional to the Fourier transform of the input ${P}_{s}\left(\xi ,\eta \right)$:

**Table 1:** Summary of the approximations required to derive the UFT expression.

No	Approximation	Validity
1	$\left|{\varvec{r}}-{{\varvec{r}}}_{0}\right|\approx f\left[1+\frac{1}{2}{\left(\frac{x-\xi }{f}\right)}^{2}+\frac{1}{2}{\left(\frac{y-\eta }{f}\right)}^{2}\right]$	Fresnel
2	$\left|{\varvec{r}}-{{\varvec{r}}}_{0}\right|\approx f$	Paraxial
3	$\mathrm{cos}\left({\varvec{n}},{\varvec{r}}-{{\varvec{r}}}_{0}\right)\approx 1$	Paraxial
4	$\frac{1}{\left|{\varvec{r}}-{{\varvec{r}}}_{0}\right|}+jk\approx jk$	Distances much larger than $\lambda$

1 $$\begin{array}{c}{P}_{O}\left(u,v\right)=\frac{j\,\,\mathrm{exp}\left(-2jkf\right)}{\lambda f}F\left\{{P}_{S}\left(\xi ,\eta \right)\right\},\end{array}$$

where the operator $\mathcal{F}$ denotes the FT, $j=\sqrt{-1}$, $k$ is the wavenumber, and $\lambda$ is the wavelength. Hence, multiplying the pressure field at the observation plane by the correction factor

2 $$\begin{array}{c}\alpha =-j\lambda f\mathrm{exp}\left(2jkf\right)\end{array}$$

theoretically yields the exact FT of the pressure field at the source plane. Hereinafter, the corrected output pressure field will be referred to as the UFT of the input.

### Design of the UFT-ACS

The operating frequency of the UFT-ACS was chosen as ${f}_{wave}=1.7$ GHz, a high ultrasonic frequency that allows for a more compact system that would be easier to integrate into CMOS-compatible chips. Both substrate layers, made of fused silica due to the material’s isotropy, have a thickness of $f=1.0886$ mm. The speed of ultrasonic waves is ${v}_{wave}=5880$ m s^−1^ in fused silica, which implies $\lambda ={v}_{wave}/{f}_{wave}=3.46$ µm.

**Figure 2:**
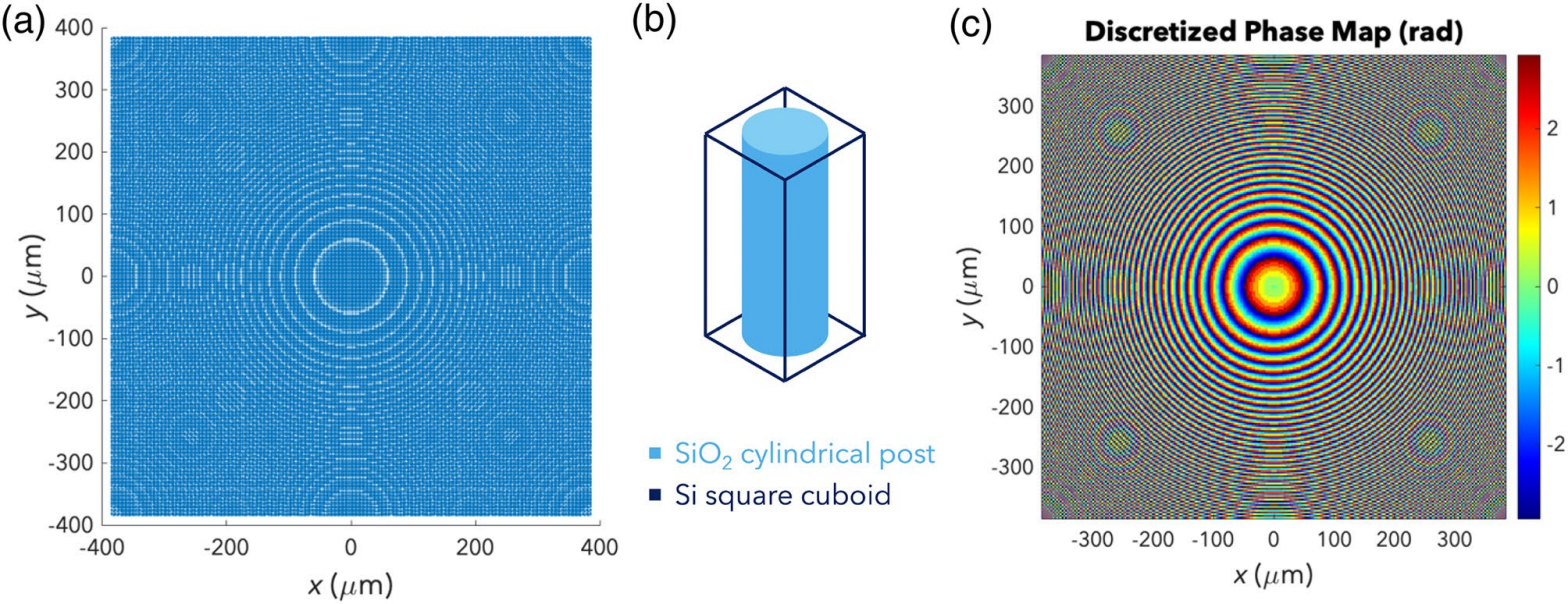
Ultrasonic metalens. (**a**) Top view of the ultrasonic metalens. (**b**) Unit cell made up of a SiO_2_ cylindrical post (light blue) embedded in a Si square cuboid (dark blue). (**c**) Discretized phase map of the metalens.

The metalens, shown in Fig. 2a, consists of many unit cells with a square cross-section of side length 3 µm (a subwavelength feature) and a thickness of ${t}_{m}=16$ µm. In Fig. 2b, each unit cell consists of a cylindrical post made of SiO_2_ embedded in Si. Theoretically, the ultrasonic metalens should have a paraboloidal phase profile

3 $$\begin{array}{c}{\phi }_{ideal}\left(x,y\right)=k\left(\frac{{x}^{2}+{y}^{2}}{2f}\right)\end{array}$$

to obtain the UFT. However, discretization is needed as there is a limited number of distinct unit cells. Thus, the metalens’ unit cells should be arranged such that the unit cell at each point has a cylindrical post with a radius corresponding to that point’s interpolated phase shift. After interpolation, the ideal phase map becomes the discretized phase map, shown in Fig. 2c.

### UFT of various input functions

**Figure 3:**
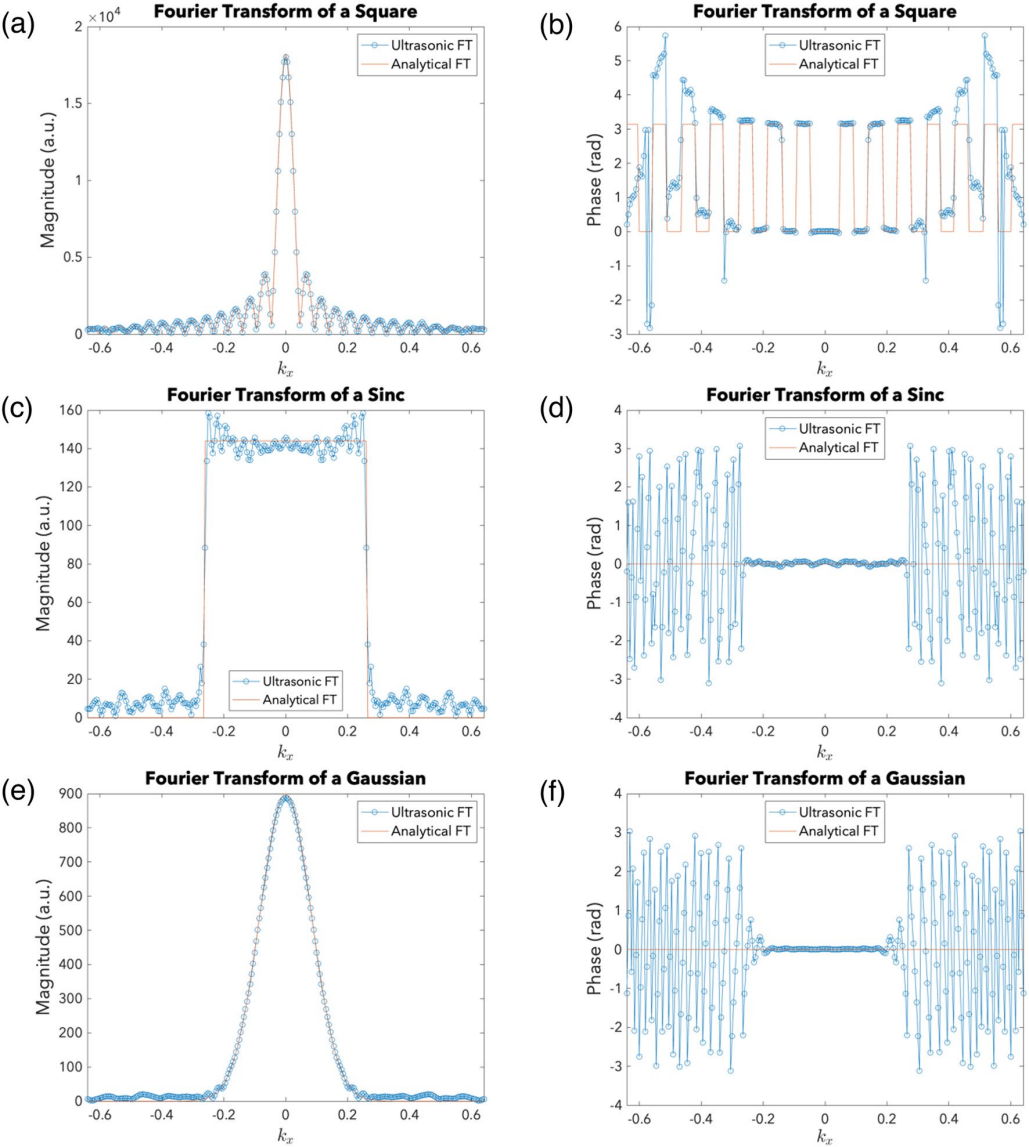
Ultrasonic Fourier Transform (UFT) Simulations. (**a**) Square: Magnitude profiles of the UFT and the analytical FT. (**b**) Square: Phase profiles of the UFT and the analytical FT. (**c**) Sinc: Magnitude profiles of the UFT and the analytical FT. (**d**) Sinc: Phase profiles of the UFT and the analytical FT. (**e**) Gaussian: Magnitude profiles of the UFT and the analytical FT. (**f**) Gaussian: Phase profiles of the UFT and the analytical FT.

A space-limited function is one whose non-zero values are contained in a finite region in the space domain, while a bandlimited function has a finite spectral width containing all spatial frequency components with non-zero magnitude values. In the context of the FT, there are three main types of functions: space-limited but not bandlimited (Type I), bandlimited but not space-limited (Type II) and neither space-limited nor bandlimited (Type III). A function cannot be both space-limited and bandlimited^39^. A square input, defined by $f\left(\xi ,\eta \right)=\mathrm{rect}\left(\xi /w\right)\mathrm{rect}\left(\eta /w\right)$ with $w=135$, was used as a sample Type I input. For Type II functions, a two-dimensional sinc function, specifically $f\left(\xi ,\eta \right)=\mathrm{sinc}\left(\xi /12\right)\mathrm{sinc}\left(\eta /12\right)$, was used as a sample input. Lastly, the two-dimensional Gaussian $f\left(\xi ,\eta \right)=\mathrm{exp}\left\{-\pi \left[{\left(\xi /\gamma \right)}^{2}+{\left(\eta /\gamma \right)}^{2}\right]\right\}$ with $\gamma =30$ was used as a sample Type III input. Referring to Fig. 3, the simulation results demonstrate that the UFT-ACS is indeed an accurate Fourier transformer.

For the sample square input, the root-mean-squared error (RMSE) after normalization is 0.9%. The UFT’s magnitude profile is in excellent agreement with the analytical FT’s magnitude profile (Fig. 3a), with only minor deviations towards the edges since the UFT expression is derived only after Fresnel and paraxial approximations are made (see Table 1). Thus, they are expected to only agree in the central region. The phase profiles (Fig. 3b) of the UFT and analytical FT only match well at the center for the same reason. Aberration due to the metalens’ discretized phase profile also contributes to the observed discrepancies in both the magnitude and phase profiles. Moreover, there is some aliasing due to bandlimiting as the square’s FT is not bandlimited.

For the sample sinc input, the RMSE after normalization is 4.6%. Figure 3c shows that there is a somewhat good agreement between the magnitude profiles of the UFT and the analytical FT. As explained by Gibbs’ phenomenon^49^ (see Supplementary Information), ripple artifacts can be observed in the UFT’s magnitude profile as a result of truncating the input sinc function—an inevitable consequence of the UFT-ACS’ finite size. Aberration due to the discretized phase profile of the metalens exacerbated the ripples and caused small lobes to be observed at the sides (see Supplementary Information). Nonetheless, the overall shape of the UFT’s magnitude profile still resembles that of the analytical FT. Figure 3d shows that the phase of the UFT and the analytical FT only match at the center, where the magnitude is significant. This can be attributed to the fact that the UFT is only achieved in the paraxial region and to the fact that the magnitude is supposedly zero but there are ripple artifacts due to truncation. The latter implies that a small error in the real and imaginary parts of the complex pressure field results in non-negligible error in the phase. Metalens aberration further contributes to the error.

For the sample Gaussian input, the RMSE after normalization is 0.4%. It is evident from Fig. 3e that the magnitude profiles of the UFT and the analytical FT agree well with each other. Granted, there are some small lobes towards the edges of the magnitude profile, which can be attributed to aberration due to the discretized phase profile of the metalens (see Supplementary Information). Be that as it may, the significant magnitude values are accurate, and the overall shape is preserved. Moreover, it is also evident from Fig. 3f that the phase profile of the UFT coincides with that of the analytical FT only at the center. This is because of the approximations required to obtain the UFT expression and aberration due to the discretized phase profile of the metalens.

Overall, for all three types of input functions, the UFT satisfactorily matches the analytical FT in both magnitude and phase (see Table 2 for summary of RMSEs). The UFT’s magnitude profile has somewhat noticeable errors towards the edges, and the UFT’s phase profile, as can be observed from Fig. 3, matches the analytical FT’s phase profile at the center and when the magnitude is significant. This is acceptable as, in general, only the significant magnitude values and their corresponding phase values are of interest in FT applications. Furthermore, the overall shapes of the UFT and analytical FT’s magnitude profiles resemble each other well, which is usually sufficient for applications.

**Table 2:** RMSE for sample input functions.

Type of Input	RMSE (%)
I	0.9
II	4.6
III	0.4

### Optimization of accuracy

Having examined the accuracy of the UFT for all three types of inputs, this section explores how the accuracy can be optimized through appropriate zero padding, truncation, and bandlimiting.

#### Zero padding

Besides the zero padding done to the pressure field array at the source plane to avoid circular convolution errors associated with FFT-based convolution^46–48^, space-limited functions must also be zero-padded within the sampled array bounds $\xi ,\eta \in [-(L-\Delta )/2,(L-\Delta )/2]$.

To study the effect of zero padding on the UFT-ACS’ accuracy, MATLAB was used to simulate the UFT calculation for numerous square input functions of different side lengths. The side length $w$ was varied incrementally from 3 to 765, inclusive. Both the UFT and the analytical FT were normalized with the maximum value of the analytical FT as 1. This is done so that a fair comparison of the errors can be made. After normalization, the root-mean-squared error (RMSE) between the magnitude pattern of the UFT and that of the analytical FT was calculated, using MATLAB’s mse and sqrt functions, for all $w$.

**Figure 4:**
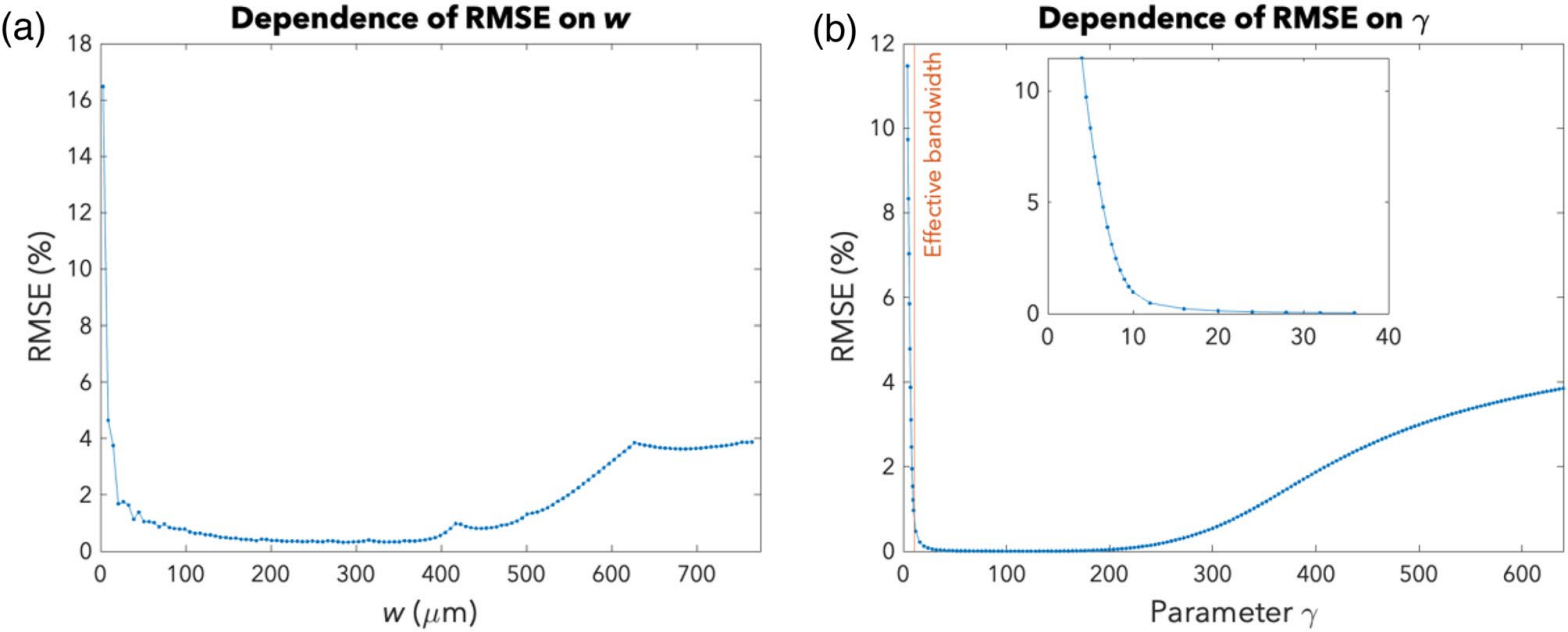
Accuracy optimization: zero padding, truncation, and bandlimiting**.** (**a**) The graph shows the dependence of the RMSE on the side length $w$ of the input square, which is inversely related to the amount of zero padding. (**b**) The graph shows the dependence of the RMSE on $\gamma$. The blue dots represent the RMSE of the simulations involving an ideal metalens. The orange vertical line indicates the value of $\gamma$ corresponding to the case wherein the sampled array just contains 98% of the total spectral power.

Figure 4a shows how the RMSE varies with the side length $w$ of the input square function, which is inversely related to the amount of zero padding. It can be observed that the error initially decreases as the width $w$ increases or as the amount of zero padding decreases. This occurs because as $w$ increases, the effective bandwidth of the input function decreases, resulting in a decrease in error due to reduced aliasing in the spatial frequency domain. The error eventually starts to increase as $w$ continues increasing. This can be attributed to the fact that the UFT is only achieved in the paraxial region. Refer to Supplementary Information for additional explanatory diagrams supporting the above analysis.

Thus, a moderate number of zeros—neither too little nor too much—must be used to pad space-limited input functions in order to accurately calculate the FT.

#### Truncation and bandlimiting

Truncation, otherwise known as windowing, refers to limiting the spatial extent of a function that is not space-limited. Analogously, bandlimiting refers to limiting the bandwidth of a function that is not bandlimited.

To study their impact on accuracy, UFT-ACS simulations were carried out for Gaussians with $\gamma$ (the parameter affecting a Gaussian’s width) varied incrementally from 4 to 640, inclusive. Both the UFT (when using an ideal metalens) and the analytical FT were normalized. After normalization, the RMSE was calculated for the results of each value of $\gamma$. The UFT with an ideal metalens was used for comparison with the analytical FT so as to eliminate the errors caused by aberration due to the discretized phase profile of the metalens and, therefore, ascertain that the error is indeed ascribed to truncation and bandlimiting.

Figure 4b shows how the RMSE varies with $\gamma$. Initially, the RMSE decreases as $\gamma$ increases. Notably, referring to the inset in Fig. 4b, the initial decrease is very steep until the orange line, which corresponds to the value of $\gamma$ for which the effective bandwidth is just contained within the sampled array bounds. However, this decreasing trend is only observed until a certain critical point, beyond which the RMSE starts to increase again.

The initial decrease occurs because as $\gamma$ increases, the effective bandwidth of the input function decreases, resulting in a greater percentage of the total spectral power being captured within the sampled array bounds. Therefore, there is a decrease in error due to reduced aliasing in the spatial frequency domain. Moreover, the paraxial approximations become more valid as $\gamma$ initially increases because if $\gamma$ is too small, the energy, which is initially highly concentrated at the centre of the space domain, becomes very spread out in the spatial frequency domain. Eventually, the RMSE stops decreasing and starts rising as $\gamma$ increases further. There are two reasons for this. Firstly, for a higher $\gamma$, the energy is spread out more in the space domain, resulting in more significant magnitudes lying outside the sampled array bounds. Thus, there is an increase in error due to undersampling in the space domain, outweighing the decrease in error due to reduced aliasing in the spatial frequency domain associated with a higher $\gamma$. Secondly, the approximations are less valid when $\gamma$ is too large because the energy, initially spread out in the space domain, becomes highly concentrated at the center of the spatial frequency domain. Supplementary Information provides additional graphs supporting the above reasoning.

Hence, to optimize the accuracy of the UFT, the parameter $\gamma$ should be neither too small nor too large such that the input function and its FT are both of moderate width and the approximations are more valid.

## Discussion

In summary, this paper presents the Ultrasonic Fourier Transform Analog Computing System (UFT-ACS), which has been demonstrated to perform FT calculations for all three types of functions to a relatively high degree of accuracy. The simulations in this study have shown the true capabilities, appropriately qualified by the limitations, of the UFT-ACS—addressing this knowledge gap in the existing literature. Optimizing the UFT’s accuracy was also explored and better understood by studying the effect of zero padding, truncation and bandlimiting.

This study’s findings are of considerable significance. Performing FT calculations faster than the electronic FFT algorithm, the UFT-ACS satisfies the growing demand for such capabilities in some applications like real-time image processing in autonomous vehicles. Existing analog computing systems also make use of the FT to perform mathematical operations, such as spatial differentiation, integration, and convolution. Thus, the UFT-ACS can also impact the broader field of wave-based analog computing. It can improve the prospects of wave-based analog computers as potential supercomputers in the future, possibly surpassing the current limitations of today’s electronic computers.

## Methods

### Ultrasonic metalens designing process

Referring to Supplementary Fig. S3, designing the metalens involves a few simple steps. Firstly, carry out unit cell simulations in order to obtain a relationship between the phase shift due to a particular unit cell and the radius of that unit cell’s cylindrical post. Secondly, obtain an array of the ideal phase map consisting of phase values at sampled points following the theoretical paraboloidal phase profile

4 $$\begin{array}{c}{\phi }_{ideal}\left(x,y\right)=k\left(\frac{{x}^{2}+{y}^{2}}{2f}\right)\end{array}$$

required to obtain the UFT expression. Thirdly, perform interpolation to the nearest available phase value from the unit cell simulations using the MATLAB function interp1. Thus, at this juncture, we have obtained the discretized phase map consisting of phase values which have a corresponding radius from the unit cell simulations. Subsequently, use the phase-to-radius mapping to obtain a radius map—an array of radius values at each sampled point. Finally, using the MATLAB function viscircles, generate a figure of the metalens comprising unit cells whose cylindrical posts have a radius corresponding to the radius at that point as per the radius map previously obtained.

### Wave propagation simulations

Exact solutions—that is, before Fresnel and paraxial approximations were applied—of the Kirchhoff-Helmholtz Integral were used to numerically simulate the propagation of ultrasonic waves through the UFT-ACS, and the results were compared with the analytical FT. The code was implemented using MATLAB.

As opposed to Finite Element Method (FEM), this semi-analytical approach is much less computationally costly and, therefore, allows for simulations involving considerably larger arrays—key to understanding the UFT-ACS’ true capabilities.

The pressure field ${\overline{P} }_{M-}(x,y)$ right in front of the metalens can be obtained by using an FFT-based convolution approach to convolve the zero-padded input pressure field array ${\overline{P} }_{S}(\xi ,\eta )$ with the convolution kernel

5 $$\begin{array}{c}{\overline{h} }_{1}\left(\xi ,\eta \right)=\frac{j\mathrm{exp}\,\,\left(-jk\sqrt{{f}^{2}+{\xi }^{2}+{\eta }^{2}}\right)}{\lambda \sqrt{{f}^{2}+{\xi }^{2}+{\eta }^{2}}}.\end{array}$$

Note that the $N\times N$ array ${\overline{P} }_{S}(\xi ,\eta )$ must be padded by at least $N-1$ zeros to avoid circular convolution errors^47^. By convention, the convolution kernel array is the same size as the zero-padded pressure field array^47^. ${\overline{P} }_{M-}(x,y)$ is then the $N\times N$ subarray at the center of the larger array produced by FFT-based convolution. The second part of the simulation involves applying the phase shift due to the discretized metalens to obtain the $N\times N$ pressure field ${\overline{P} }_{M+}(x,y)$ right behind the metalens. It can be obtained by the element-wise multiplication of the $N\times N$ array ${\overline{P} }_{M-}\left(x,y\right)$ and the $N\times N$ array $\mathrm{exp}\left(i{\overline{\phi }}_{discretized}\right)$, where ${\overline{\phi }}_{discretized}$ is the discretized phase profile of the metalens after interpolating each phase shift value to the closest available phase data from the unit cell simulations. Subsequently, the $N\times N$ output pressure field array ${\overline{P} }_{O}(u,v)$ is obtained by using FFT-based convolution to convolve the zero-padded array ${\overline{P} }_{M+}(x,y)$ with the convolution kernel

6 $$\begin{array}{c}{\overline{h} }_{2}\left(x,y\right)=\left(\frac{1}{\sqrt{{f}^{2}+{x}^{2}+{y}^{2}}}+jk\right)\frac{f\mathrm{exp}\left(-jk\sqrt{{f}^{2}+{x}^{2}+{y}^{2}}\right)}{2\pi \left({f}^{2}+{x}^{2}+{y}^{2}\right)}.\end{array}$$

Finally, the UFT result is obtained by multiplying the $N\times N$ pressure field array ${\overline{P} }_{o}(u,v)$ at the observation plane by the correction factor $\alpha =-j\lambda f\mathrm{exp}\left(2jkf\right)$.

See Supplementary Information for the full derivation, which is partly the independent work of the authors.

### Simulation parameters

Choosing the appropriate values of the simulation parameters is important as this affects the accuracy of the UFT as well as the design of the physical system to be fabricated.

The selection of simulation parameters is a three-step process. Firstly, as required by convolution^47^, the spacing $\Delta$ between the sampled points of the pressure fields in the source, metalens and observation planes must be the same as the spacing between adjacent metalens unit cells. Secondly, depending on the specific application for which the UFT-ACS is being used, an appropriate length $L$ for the source, metalens and observation planes must be chosen. Zero padding must be moderate; truncation and bandlimiting must be done appropriately such that the significant space and spatial frequency components are within the sampled array bounds. Thirdly, the focal length $f$ must satisfy

7 $$\begin{array}{c}f\ge \sqrt{{\left[\frac{2\left(L-\Delta \right)\Delta }{\lambda }\right]}^{2}-{\left(L-\Delta \right)}^{2}},\end{array}$$

which is derived from a consideration of the sampling requirements of the convolution kernels’ exponential phase term^36,44–48^. A detailed explanation for each step is offered in the Supplementary Information. Table 3 summarizes the values of the simulations’ parameters.

**Table 3:** Summary of parameter values.

Parameter	Value
$\Delta$	3 µm
$L$	771 µm
${f}_{wave}$	1.7 GHz
${v}_{wave}$	5880 m s^-1^
$f$	1088.6 µm

## Supplementary Information

Supplementary Information.

## Data Availability

The datasets generated during and/or analyzed during the current study are available from the corresponding author on reasonable request.
