# Supplementary material for: A metalens-based analog computing system for ultrasonic Fourier transform calculations
Source: Sci Rep. 2022 Oct 12;12:17124. doi: 10.1038/s41598-022-21753-9 (PMC9556540; doi:10.1038/s41598-022-21753-9)
Supplement: Supplementary file 1 — Supplementary Information. [file 41598_2022_21753_MOESM1_ESM.docx]

**Supplementary Information for**

***A metalens-based analog computing system for ultrasonic Fourier transform calculations***

**Robert Frederik Uy**^1,*^ **and Bui Viet Phuong**^2^_­_

^1^Hwa Chong Institution, Singapore, 269734, Singapore

^2^A*STAR Institute of High Performance Computing, Electronics and Photonics Department, Singapore, 138632, Singapore

*Corresponding Author: robertfrederikduy@gmail.com

**Supplementary Note: Scalar Diffraction Theory**

**Kirchhoff-Helmholtz Integral Theorem**

The Kirchhoff-Helmholtz Integral Theorem (KHIT) states that

$$\begin{aligned} P\left( \boldsymbol{r} \right)=\int_{S=S_{o}+S_{h}} \left[ G\left( \boldsymbol{r} | \boldsymbol{r}_{0} \right)\frac{\partial P\left( \boldsymbol{r}_{0} \right)}{\partial n\left( \boldsymbol{r}_{0} \right)}-P\left( \boldsymbol{r}_{0} \right)\frac{\partial G\left( \boldsymbol{r} | \boldsymbol{r}_{0} \right)}{\partial n\left( \boldsymbol{r}_{0} \right)} \right]dS\left( \boldsymbol{r}_{0} \right),\#\left( S1 \right) \end{aligned}$$

where $P(\boldsymbol{r})$ is the acoustic pressure complex amplitude at point $\boldsymbol{r}$, $\boldsymbol{n}(\boldsymbol{r}_{0})$ is a unit vector normal to the source plane’s surface and $G(\boldsymbol{r}|\boldsymbol{r}_{0})$ is a Green’s function for harmonic waves^36,52-55^. The integral in Supplementary Eq. S1 is evaluated over the composite surface $S$, which consists of the active surface $S_{o}$ of the source plane and the closing surface $S_{h}$. Supplementary Fig. S1 is a diagram showing the composite surface $S$, which is the domain of integration, and the relevant vectors in the integral.

Evaluating the integral in Supplementary Eq. S1 to model the propagation of ultrasonic waves requires the application of appropriate boundary conditions. In this study, two boundary conditions were considered: (i) the source is embedded in a half-space with an acoustically hard boundary and (ii) the source is embedded in a half-space with an acoustically soft boundary.

|  |
| --- |
| **Supplementary Figure S1. Acoustic radiation source embedded in a half-space.** The figure shows the source embedded in a half-space. The dark blue region, labelled $S_{o}$, is the active suface of the source plane. The surrounding light blue region and the dashed lines (which represent the boundary at infinity) form the closing surface $S_{h}$. Adapted from Ref. 52. |

**Source is embedded in a half-space with an acoustically hard boundary**

For the first boundary condition, the Green’s function to be considered is

$$\begin{aligned} G\left( \boldsymbol{r} | \boldsymbol{r}_{0} \right)=-\frac{\exp\left( -jk\left| \boldsymbol{r}-\boldsymbol{r}_{0} \right| \right)}{2\pi\left| \boldsymbol{r}-\boldsymbol{r}_{0} \right|},\#\left( S2 \right) \end{aligned}$$

whose normal derivative $\partial G/\partial n$ is zero^52^. Moreover, in this case, the normal pressure derivative $\partial P/\partial n$ nullifies on the closing surface $S_{h}$. Thus, in the case of a plane wave with amplitude $P(\boldsymbol{r}_{0})$, Supplementary Eq. (S1) can be expressed as

$$\begin{aligned} P\left( \boldsymbol{r} \right)=\frac{j}{\lambda}\int_{S_{o}} P\left( \boldsymbol{r}_{0} \right)\frac{\exp\left( -jk\left| \boldsymbol{r}-\boldsymbol{r}_{0} \right| \right)}{\left| \boldsymbol{r}-\boldsymbol{r}_{0} \right|}dS\left( \boldsymbol{r}_{0} \right),\#\left( S3 \right) \end{aligned}$$

where $\lambda$ is the wavelength of the emitted acoustic wave^52^.

**Source is embedded in a half-space with an acoustically soft boundary**

For the second boundary condition, the Green’s function vanishes^52^ but its normal derivative is given by

$$\begin{aligned} \frac{\partial G\left( \boldsymbol{r} | \boldsymbol{r}_{0} \right)}{\partial n\left( \boldsymbol{r}_{0} \right)}=-\left( \frac{1}{\left| \boldsymbol{r}-\boldsymbol{r}_{0} \right|}+jk \right)\frac{\exp\left( -jk\left| \boldsymbol{r}-\boldsymbol{r}_{0} \right| \right)}{2\pi\left| \boldsymbol{r}-\boldsymbol{r}_{0} \right|}\cos\left( \boldsymbol{n},\boldsymbol{r}-\boldsymbol{r}_{0} \right),\#\left( S4 \right) \end{aligned}$$

which is the exact version, independently derived by the authors, of the approximate expression given in Ref. 52. In this case, the pressure $P$ on the closing surface $S_{h}$ is zero, so Supplementary Eq. (S1) becomes

$$\begin{aligned} P\left( \boldsymbol{r} \right)=\frac{1}{2\pi}\int_{S_{o}} P\left( \boldsymbol{r}_{0} \right)\left( \frac{1}{\left| \boldsymbol{r}-\boldsymbol{r}_{0} \right|}+jk \right)\times\frac{\exp\left( -jk\left| \boldsymbol{r}-\boldsymbol{r}_{0} \right| \right)}{\left| \boldsymbol{r}-\boldsymbol{r}_{0} \right|}\cos\left( \boldsymbol{n},\boldsymbol{r}-\boldsymbol{r}_{0} \right)dS\left( \boldsymbol{r}_{0} \right),\#\left( S5 \right) \end{aligned}$$

which, upon applying the approximations $jk+\left( \left| \boldsymbol{r}-\boldsymbol{r}_{0} \right| \right)^{-1}\approx jk$ and $\cos\left( \boldsymbol{n},\boldsymbol{r}-\boldsymbol{r}_{0} \right)\approx1$, simplifies into

$$\begin{aligned} P\left( \boldsymbol{r} \right)=\frac{j}{\lambda}\int_{S_{o}} P\left( \boldsymbol{r}_{0} \right)\frac{\exp\left( -jk\left| \boldsymbol{r}-\boldsymbol{r}_{0} \right| \right)}{\left| \boldsymbol{r}-\boldsymbol{r}_{0} \right|}dS\left( \boldsymbol{r}_{0} \right).\#\left( S6 \right) \end{aligned}$$

**Approximate Solution to the KHIT**

Using the Fresnel approximation

$$\begin{aligned} \left| \boldsymbol{r}-\boldsymbol{r}_{0} \right|=f\sqrt{1+\left( \frac{x-\xi}{f} \right)^{2}+\left( \frac{y-\eta}{f} \right)^{2}}\approx f\left[ 1+\frac{1}{2}\left( \frac{x-\xi}{f} \right)^{2}+\frac{1}{2}\left( \frac{y-\eta}{f} \right)^{2} \right]\#\left( S7 \right) \end{aligned}$$

in the numerator and the paraxial approximation

$$\begin{aligned} \left| \boldsymbol{r}-\boldsymbol{r}_{0} \right|\approx f\#\left( S8 \right) \end{aligned}$$

in the denominator, both Supplementary Eqs. (S3) and (S6) can be re-written as

$$\begin{aligned} P\left( \boldsymbol{r} \right)=\frac{j}{\lambda f}\int_{S_{o}} P\left( \boldsymbol{r}_{0} \right)\exp\left\{ -jkf\left[ 1+\frac{1}{2}\left( \frac{x-\xi}{f} \right)^{2}+\frac{1}{2}\left( \frac{y-\eta}{f} \right)^{2} \right] \right\}dS\left( \boldsymbol{r}_{0} \right),\#\left( S9 \right) \end{aligned}$$

which is the approximate expression for the pressure field at a point in the half-space with position vector $\boldsymbol{r}$**.**

**Supplementary Note: Ultrasonic Fourier Transform Mathematical Formulation**

To mathematically derive the overall UFT expression for the UFT-ACS, the entire wave propagation process will be analysed in three parts: (i) Fresnel diffraction from the source plane to the plane right in front of the metalens, (ii) phase shift due to the metalens and (iii) Fresnel diffraction from the plane right behind the metalens to the observation plane.

Suppose, as shown in Supplementary Fig. S2, that $\left( \xi,\eta\right)$ are the coordinates of any point on the source plane, $\left( x,y \right)$ are the coordinates of any point on the metalens plane, and $\left( u,v \right)$ are the coordinates of any point on the observation plane.

|  |
| --- |
| **Supplementary Figure S2. Working principle of UFT-ACS.** The input $P_{S}(\xi,\eta)$ at the source plane propagates to the metalens plane through Fresnel diffraction and becomes $P_{M-}(x,y)$. It then undergoes a phase shift due to the metalens, resulting in the pressure field $P_{M+}(x,y)$. Finally, the wave once again propagates through Fresnel diffraction, yielding a pressure field $P_{O}(u,v)$ at the observation plane. To obtain the UFT, the metalens plane must be a distance $f$ away from both the source and observation planes. |

Firstly, the first boundary condition (acoustically hard boundary) will be applied to the KHIT for Fresnel diffraction from the source plane to the metalens. Thus, the pressure field right in front of the metalens is given by

$$\begin{aligned} P_{M-}\left( x,y \right)=\frac{j\exp\left[ -jk\left( f+\frac{x^{2}+y^{2}}{2f} \right) \right]}{\lambda f}\iint_{-\infty}^{\infty} P_{S}\left( \xi,\eta\right)\exp\left[ -jk\left( \frac{\xi^{2}+\eta^{2}}{2f} \right) \right]\exp[-j\left( k_{x}\xi+k_{y}\eta) \right]d\xi d\eta,\#\#\left( S10 \right) \end{aligned}$$

where $P_{S}\left( \xi,\eta\right)$ is the pressure field at the source plane and $k_{x}$ and $k_{y}$ are the spatial frequencies in the Fourier domain defined by

$$\begin{aligned} k_{x}=-\frac{kx}{f}, k_{y}=-\frac{ky}{f}.\#\left( S11 \right) \end{aligned}$$

Using the definition of the Fourier transform, we can re-write Supplementary Eq. (S10) as

$$\begin{aligned} P_{M-}\left( x,y \right)=\frac{jP_{1}\exp\left[ -jk\left( f+\frac{x^{2}+y^{2}}{2f} \right) \right]}{\lambda f}\mathcal{F}\left\{ P_{S}\left( \xi,\eta\right)\exp\left[ -jk\left( \frac{\xi^{2}+\eta^{2}}{2f} \right) \right] \right\}.\#\left( S12 \right) \end{aligned}$$

Another way to express Supplementary Eq. (S10) is in the form of a convolution equation

$$\begin{aligned} P_{M-}\left( x,y \right)=\frac{j\exp\left( -jkf \right)}{\lambda f}\iint_{-\infty}^{\infty} P_{S}\left( \xi,\eta\right)\exp\left\{ -j\frac{k}{2f}\left[ \left( x-\xi\right)^{2}+\left( y-\eta\right)^{2} \right] \right\} d\xi d\eta,\#\left( S13 \right) \end{aligned}$$

where the convolution kernel, also known as the impulse response, is given by

$$\begin{aligned} h\left( \xi,\eta\right)=\frac{j\exp\left( -jkf \right)}{\lambda f}\exp\left[ -j\frac{k}{2f}\left( \xi^{2}+\eta^{2} \right) \right].\#\left( S14 \right) \end{aligned}$$

Using the Convolution Theorem, we obtain

$$\begin{aligned} \mathcal{F}\left\{ P_{M-}\left( x,y \right) \right\}\mathcal{=F}\left\{ P_{S}\left( \xi,\eta\right) \right\}\exp\left( -jkf \right)\exp\left[ \frac{j\lambda f}{4\pi}\left( k_{x}^{2}+k_{y}^{2} \right) \right].\#\left( S15 \right) \end{aligned}$$

Secondly, in order to eventually obtain the UFT expression, the metalens should cause a paraboloidal phase shift, so the pressure field at the plane right behind the metalens is given by

$$\begin{aligned} P_{M+}\left( x,y \right)=P_{M-}\left( x,y \right)\exp\left[ jk\left( \frac{x^{2}+y^{2}}{2f} \right) \right].\#\left( S16 \right) \end{aligned}$$

Thirdly, the second boundary condition (acoustically soft boundary) will be applied to the KHIT for Fresnel diffraction from the metalens to the observation plane. Thus, the pressure field at the observation plane is given by

$$\begin{aligned} P_{O}\left( u,v \right)=\frac{j\exp\left[ -jk\left( f+\frac{u^{2}+v^{2}}{2f} \right) \right]}{\lambda f}\iint_{-\infty}^{\infty} P_{M+}\left( x,y \right)\exp\left[ -jk\left( \frac{x^{2}+y^{2}}{2f} \right) \right]\exp[-j\left( k_{x}x+k_{y}y) \right]dxdy.\#\left( S17 \right) \end{aligned}$$

Substituting Supplementary Eq. (S16) into Supplementary Eq. (S17) yields

$$\begin{aligned} P_{O}\left( u,v \right)=\frac{j\exp\left[ -jk\left( f+\frac{u^{2}+v^{2}}{2f} \right) \right]}{\lambda f}\mathcal{F}\left\{ P_{M-}(x,y) \right\}.\#\left( S18 \right) \end{aligned}$$

which, using Supplementary Eq. (S15), can be re-written as

$$\begin{aligned} P_{O}\left( u,v \right)=\frac{j\exp\left( -2jkf \right)}{\lambda f}\mathcal{F}\left\{ P_{S}\left( \xi,\eta\right) \right\},\#\left( S19 \right) \end{aligned}$$

which is proportional to the Fourier transform of the pressure field at the source plane. Hence, it has been shown that by simply multiplying the pressure field at the observation plane by the correction factor

$$\begin{aligned} \alpha=-j\lambda f\exp\left( 2jkf \right),\#\left( S20 \right) \end{aligned}$$

the Fourier transform of the pressure field at the source plane can be obtained.

**Supplementary Figure S3**

|  |
| --- |
| **Supplementary Figure S3. Metalens Designing Process.** The flowchart shows the five-step process of designing a metalens. This process yields the full top view of the metalens designed for the UFT-ACS. |

**Supplementary Note: Simulation Parameters**

In order to obtain accurate results, certain sampling conditions must be met. Firstly, the input function’s space components with significant magnitude values must be within the sampled array bounds $\xi,\eta\in[-(L-\Delta\xi)/2,(L-\Delta\xi)/2]$. Ideally, the function is space-limited such that all of its non-zero values can fit within the aforementioned sampled array bounds. Secondly, the Whittaker-Shannon Sampling Theorem must be satisfied. It states that the maximum spacing of the sampled input function is given by the Nyquist rate:

$$\begin{aligned} \Delta x\leq\frac{1}{B_{x}}, \Delta y\leq\frac{1}{B_{y}},\#\left( S21 \right) \end{aligned}$$

where $B_{x}$ and $B_{y}$ are the function’s bandwidth in the $x$ and $y$ directions^36^. For functions which are not bandlimited, the ‘effective bandwidth’, which encompasses most of the input function’s significant spatial frequency values (typically 98% of the spectral power), can be used instead^44^. Thirdly, there are some exponential phase terms which are space-limited to a finite interval, and these must be sampled adequately according to

$$\begin{aligned} \Delta x\left| \frac{\partial\phi}{\partial x} \right|_{max}\leq\pi, \Delta y\left| \frac{\partial\phi}{\partial y} \right|_{max}\leq\pi,\#\left( S22 \right) \end{aligned}$$

where $\phi$ is the phase expression in the exponential phase term being sampled^45^. In the UFT simulations, there are two exponential phase terms being sampled, namely

$$\begin{aligned} \exp\left( -jk\sqrt{x^{2}+y^{2}+f^{2}} \right), \exp\left[ -jk\left( \frac{x^{2}+y^{2}}{2f} \right) \right].\#\left( S23 \right) \end{aligned}$$

The second is sampled by an $N\times N$ array. If $N$ is odd (which is the case in this study, then the first one is sampled by a $\left( 2N-1 \right)\times(2N-1)$ array. Thus, we obtain two conditions on the possible focal lengths $f$. The first exponential phase term requires that

$$\begin{aligned} f\geq\sqrt{\left[ \frac{2\left( L-\Delta x \right)\Delta x}{\lambda} \right]^{2}-\left( L-\Delta x \right)^{2}},\#\left( S24 \right) \end{aligned}$$

whereas the second one, which is sampled by an $N\times N$ array, requires

$$\begin{aligned} f\geq\frac{(L-\Delta x)\Delta x}{\lambda}.\#\left( S25 \right) \end{aligned}$$

Therefore, we obtain the overall condition

$$\begin{aligned} f\geq\sqrt{\left[ \frac{2\left( L-\Delta x \right)\Delta x}{\lambda} \right]^{2}-\left( L-\Delta x \right)^{2}},\#\left( S26 \right) \end{aligned}$$

which gives the minimum value of $f$ that satisfies both conditions. Deciding on the exact value of $f$ involves balancing between two trade-offs. On the one hand, a smaller $f$ allows for a more compact UFT-ACS. A larger $f$, on the other hand, will lead to a larger central region of the observation plane for which the Fresnel and paraxial approximations are valid. In this study, the minimum possible $f$ was chosen.

**Supplementary Note: Ultrasonic Fourier Transform of a Square**

|   (a)  (c) |   (b) |
| --- | --- |
|  |   (d) |
|   (e) |   (f) |
| x  (g) |   (h) |
| **Supplementary Figure S4. Fourier Transform of a Square.** (a) Magnitude pattern at the source plane. (b) Phase pattern at the source plane. (c) Magnitude pattern of the UFT. (d) Magnitude pattern of the analytical FT. (e) Phase pattern of the UFT. (f) Phase pattern of the analytical FT. (g) Magnitude profiles of the UFT (blue circles) vs the analytical FT (orange line). (h) Phase profiles of the UFT (blue circles) vs the analytical FT (orange line). | |

**Supplementary Note: Ultrasonic Fourier Transform of a Sinc**

|   (a) |   (b) |
| --- | --- |
|   (c) |   (d) |
|   (e) |   (f) |
|   (g) |   (h) |
| **Supplementary Figure S5. Fourier Transform of a Sinc.** (a) Magnitude pattern at the source plane. (b) Phase pattern at the source plane. (c) Magnitude pattern of the UFT. (d) Magnitude pattern of the analytical FT. (e) Phase pattern of the UFT. (f) Phase pattern of the analytical FT. (g) Magnitude profiles of the UFT (blue circles) vs the analytical FT (orange line). (h) Phase profiles of the UFT (blue circles) vs the analytical FT (orange line). | |

In the main text, Gibbs’ phenomenon was used to explain the ripple artifacts. Here, further elaboration is provided for greater clarity. The Fourier series expansion typically only fits the actual function well for continuous functions as the series is unable to produce discontinuous functions. At discontinuities, the function’s Fourier series representation will overshoot its value^49^. Thus, since the Fourier transform of a two-dimensional sinc function is a two-dimensional rect function, which has discontinuities, the UFT of the function will also overshoot the analytical FT and have some ripple artifacts. Only in the unrealistic case of having an infinitely large UFT-ACS will the ripple artifacts be eliminated. See Supplementary Fig. S6 for a visualisation of Gibbs’ phenomenon.

Furthermore, it was stated in the main text that the ripple artifacts as explained by Gibbs’ phenomenon were exacerbated by aberration due to the discretized phase profile of the metalens. Supplementary Fig. S7 shows a comparison between the UFT results when an ideal metalens is used and the results when a discretized metalens is used.

|  |  |  |
| --- | --- | --- |
| **Supplementary Figure S6. Gibbs’ Phenomenon.** The figure shows the ripple artifacts associated with Gibbs’ Phenomenon as a result of truncation. By increasing the number of terms of the Fourier series (or by including more frequency components), the resultant curve resembles the infinite Fourier series more. |  | **Supplementary Figure S7. Comparison between UFT with an Ideal Metalens and UFT with a Discretized Metalens.** The figure shows the output of the UFT when an ideal metalens (blue circles) is used and when a discretized metalens (black dots) is used. The analytical FT (orange line) is also included for comparison purposes. |

**Supplementary Note: Ultrasonic Fourier Transform of a Gaussian**

|   (a) |   (b) |
| --- | --- |
|   (c) |   (d) |
|   (e) |   (f) |
|   (g) |   (h) |
| **Supplementary Figure S8. Fourier Transform of a Gaussian.** (a) Magnitude pattern at the source plane. (b) Phase pattern at the source plane. (c) Magnitude pattern of the UFT. (d) Magnitude pattern of the analytical FT. (e) Phase pattern of the UFT. (f) Phase pattern of the analytical FT. (g) Magnitude profiles of the UFT (blue circles) vs the analytical FT (orange line). (h) Phase profiles of the UFT (blue circles) vs the analytical FT (orange line). | |

As stated in the main text, there are small lobes towards the edges of the magnitude profile, which can be attributed to aberration due to the discretized phase profile of the metalens. In Supplementary Fig. S9, this explanation is proven by comparing the UFT’s magnitude profile when an ideal metalens is used and that when a discretized metalens is used.

|  |
| --- |
| **Supplementary Figure S9. Comparison between UFT with an Ideal Metalens and UFT with a Discretized Metalens.** The figure shows the output of the UFT when an ideal metalens (blue circles) is used and when a discretized metalens (black dots) is used. The analytical FT (orange line) is also included for comparison purposes. |

**Supplementary Note: Zero Padding**

It has been established in the main text that the RMSE initially decreases as $w$ increases [Supplementary Fig. S10(a)] because of reduced error due to aliasing in the spatial frequency domain. This occurs since the function’s effective bandwidth decreases as $w$ increases leading to reduced undersampling in the spatial frequency domain. Indeed, this can be observed by comparing Supplementary Figs. S10(b) and S10(c). Referring to Supplementary Fig. S10(b), the FT of a square with $w=9$ is severely undersampled as the sampled array bounds only captures a part of the main lobe, which means that a large portion of the spatial frequency components with significant magnitude are not sampled. In contrast, the FT of a square with $w=200$ [Supplementary Fig. S10(c)] is well-sampled as most of the significant spatial frequency components are captured within $k_{x},k_{y}\in[-(L-\Delta x)k/2f,(L-\Delta x)k/2f]$.

Furthermore, it was stated in the main text that the reason behind the eventual increase in RMSE as $w$ further increases [Supplementary Fig. S10(a)] is that the UFT is only achieved in the paraxial region. The mathematical basis for this has been discussed in the main text with the approximations explicitly outlined in Table 1. One can observe from Supplementary Figs. S10(a) and S10(d) that this is indeed true. Supplementary Fig. S10(a) shows that if the exact convolution kernel is used, the RMSE rises. On the other hand, if the approximated convolution kernel (the convolution kernels with the approximations required for UFT made) is used, the RMSE simply continues to fall. Supplementary Fig. S10(d) further corroborates the given explanation as the paraxial approximation is very clearly violated by the fact that the energy, which is initially widely spread out in the space domain, becomes highly concentrated at the centre of the spatial frequency domain.

|   (a) |   (b) |
| --- | --- |
|   (c) |   (d) |
| **Supplementary Figure S10. Zero Padding.** (a) Dependence of the RMSE on $w$. The parameter $w$ was varied from 3 to 765, and the corresponding RMSE was calculated. The blue dots represent the RMSE when the exact convolution kernel is used. The orange circles represent the RMSE when the approximate convolution kernel is used. (b) Magnitude profiles of the input square function with $w=9$ (blue) and its analytical FT (orange). (c) Magnitude profiles of the input square function with $w=200$ (blue) and its analytical FT (orange). (d) Magnitude profiles of the input square function with $w=720$ (blue) and its analytical FT (orange). For Figs. A10(b) to A10(d), the left and bottom axes coloured blue correspond to the blue-coloured input function, and the right and top axes coloured orange correspond to the orange-coloured analytical FT. | |

**Supplementary Note: Truncation and Bandlimiting**

In the main text, it was explained that the RMSE initially decreases as $\gamma$ increases [Supplementary Fig. S11(a)] due to reduced aliasing in the spatial frequency domain resulting from the decrease in the function’s effective bandwidth, just like for the square function in the Supplementary Note on Zero Padding. This is shown in Supplementary Figs. S11(b) and S11(c). The FT of a Gaussian with $\gamma=4$ is extremely undersampled as a sizeable proportion of the significant spatial frequency values are located outside the sampled array bounds, as can be seen in Supplementary Fig. S11(b). Eventually, as $\gamma$ further increases, the RMSE stops decreasing and starts increasing, as shown in Supplementary Fig. S11(a). This can be attributed to undersampling in the space domain. Indeed, this can be deduced from Supplementary Fig. S11(d), which shows that a large percentage of the function’s significant values in the space domain lie outside of the sampled array bounds. The two-part trend (decreasing then increasing) is also ascribed to the fact that the UFT is only achieved in the paraxial region. When $\gamma$ is too small or too large, the paraxial approximation is violated. Supplementary Fig. S11(b) shows that if $\gamma$ is too small, the energy, initially highly concentrated at the centre of the space domain, becomes very spread out in the spatial frequency domain. Similarly, Supplementary Fig. S11(d), which shows that if $\gamma$ is too large, the energy, which is initially more spread out in the space domain, becomes very concentrated at the centre in the spatial frequency domain.

In contrast, the Gaussian with $\gamma=160$ and its FT are both well-sampled, as shown in Supplementary Fig. S11(c). Moreover, the paraxial approximation is valid. For these reasons, the RMSE is very low (0.27%) for $\gamma=160$. This further corroborates the explanations provided for the observed discrepancies between the UFT and the analytical FT.

|   (a) |   (b) |
| --- | --- |
|   (c) |   (d) |
| **Supplementary Figure S11. Truncation and Bandlimiting.** (a) Dependence of the RMSE on $\gamma$. The parameter $\gamma$ was incrementally varied from 4 to 640, and the corresponding RMSE was calculated. The blue dots represent the RMSE of the UFT involving an ideal metalens. The orange vertical line indicates the value of $\gamma$ corresponding to the case wherein the array just contains the effective bandwidth (98% of total spectral power). (b) Magnitude profiles of the input Gaussian function with $\gamma=4$ (blue) and its analytical FT (orange). (c) Magnitude profiles of the input Gaussian function with $\gamma=160$ (blue) and its analytical FT (orange). (d) Magnitude profiles of the input Gaussian function with $\gamma=640$ (blue) and its analytical FT (orange). For Figs. A11(b) to A11(d), the left and bottom axes coloured blue correspond to the blue-coloured input function, and the right and top axes coloured orange correspond to the orange-coloured analytical FT. | |
